# *Burkholderia pseudomallei* biofilm phenotypes confined but surviving in neutrophil extracellular traps of varying appearance

**DOI:** 10.3389/fimmu.2022.926788

**Published:** 2022-08-18

**Authors:** Muthita Khamwong, Supranee Phanthanawiboon, Kanin Salao, Sorujsiri Chareonsudjai

**Affiliations:** ^1^ Department of Microbiology, Faculty of Medicine, Khon Kaen University, Khon Kaen, Thailand; ^2^ Research and Diagnostic Center for Emerging Infectious Disease (RCEID), Khon Kaen University, Khon Kaen, Thailand

**Keywords:** neutrophil extracellular traps, aggregated NETs, spiky NETs, cloudy NETs, *Burkholderia pseudomallei*, biofilm, neutrophil, confined but survive

## Abstract

Melioidosis is a fatal infectious disease caused by *Burkholderia pseudomallei*. Complications following treatment are usually due to antibiotic resistance and relapse is mainly caused by *B. pseudomallei* biofilm. Although the release of neutrophil extracellular traps (NETs) is crucial to capture and eliminate bacterial pathogens, to date response of NETs to *B. pseudomallei* biofilm is poorly understood. Here we compare the NETs produced by neutrophils in response to *B. pseudomallei* H777 (a biofilm-producing strain containing the *bpsl0618* gene), a biofilm-defect strain lacking this gene (*B. pseudomallei* M10) and a *bpsl0618* biofilm-complemented strain, *B. pseudomallei* C17, in which function of *bpsl0618* was restored. Co-cultivation of these strains with healthy human neutrophils at MOI 10 with or without cytochalasin D demonstrated that H777 significantly resisted neutrophil-mediated killing and non-phagocytotic mechanisms compared to M10 (*p* < 0.0001). Three distinct morphotypes of NETs were seen: “aggregated”, “spiky” and “cloudy”. These were induced in different proportions by the different bacterial strains. All types of NETs were shown to confine all *B. pseudomallei* strains. Strains H777 and C17 could stimulate production of twice as much extracellular DNA (234.62 ng/mL and 205.43 ng/mL, respectively) as did M10 (111.87 ng/mL). Cells of H777 and C17 were better able to survive in the presence of neutrophil killing mechanisms relative to M10 (*p* < 0.0001) and NET formation (*p* < 0.0001 and 0.05). These findings suggest that NET stimulation was insufficient to eradicate *B. pseudomallei* H777 and C17 despite their possession of *bpsl0618*, a sugar-transferase gene associated with biofilm formation ability. Our findings demonstrate that *B. pseudomallei* biofilm phenotype may be a key factor in assisting pathogens to escape killing by neutrophils. This work provides a better understanding of how *B. pseudomallei* biofilm-associated infections induce and survive NET formation, resulting in bacterial persistence and increased severity of disease.

## Introduction

*Burkholderia pseudomallei* is the causative agent of fatal infectious melioidosis. The presence of this bacterial pathogen in soil and water in endemic regions, particularly in Southeast Asia and northern Australia, is correlated with high numbers of diagnosed cases (1). The global incidence of melioidosis has been increasing, with nearly 165,000 cases and close to 90,000 deaths per year (2), including 2,800 deaths in Thailand (3). In patients, *B. pseudomallei* infection often manifests as multiple abscesses, pneumonia, septicemia and can be chronic depending on the route of infection, host immune performance, bacterial strain and load (1, 2, 4). This bacterium can be transmitted to humans *via* aerosol inhalation, ingestion and inoculation through skin injuries associated with activities of daily living (5–7). The molecular and cellular basis of pathogenesis, virulence factors and mechanisms of immune evasion that allow *B. pseudomallei* to be such an effective opportunistic pathogen have been reviewed (2, 8). However, the ways in which bacterial interaction with the immune system result in diverse disease outcomes remain to be elucidated.

Relapsing melioidosis can be a consequence of poor patient compliance with treatment regimes, has a high mortality rate (9) and is correlated with biofilm*-*associated *B. pseudomallei* infection (10). *Burkholderia pseudomallei* cells growing as biofilms are sheltered as microcolonies within extracellular polymeric substances containing proteins (11), exopolysaccharide (12) and eDNA (13) both *in vivo* and *in vitro* (12, 14). As biofilm, *B. pseudomallei* cells are effectively protected from penetration of antimicrobial agents, resulting in high-level antibiotic resistance (15, 16). Such bacterial biofilms not only habitually evade host immune defenses (17, 18) but also alter host immune responses (19), thus changing the balance between host and pathogen and contributing to the establishment of chronic infections. Several studies have attempted to understand the role of *B. pseudomallei* biofilm in the melioidosis disease process. In 2005, *B. pseudomallei* M10, a biofilm-defective mutant produced by transposon insertion into the sugar-transferase gene *bpsl0618* was constructed from the biofilm wild type H777 (20). Upregulation of *bpsl0618* increases the expression of polysaccharides for development of *B. pseudomallei* biofilm architecture (21). It appears that *bpsl0618* is crucial for *B. pseudomallei* biofilm formation. A third strain, derived from M10, is C17, in which the function of *bpsl0618* has been restored (14). The relevance of *B. pseudomallei bpsl0618* to biofilm formation, pathogenesis in alveolar epithelial cells has been established. Planktonic *B. pseudomallei* H777 cells are more able than those of M10 to attach to and invade host cells leading apoptosis and production of proinflammatory cytokines in the human lung epithelial cell line, A549. The biofilm complemented strain, C17 restored the biofilm formation, invasion ability, apoptosis induction and cytokines response (14). Additionally, the quantity of eDNA associated with C17 biofilm biomass was comparable to the parental strain, H777 (13). Many hypotheses regarding effects of *B. pseudomallei* biofilm on human innate immune cells appeared to be ill-defined.

Neutrophils are key innate immune cells for eradicating bacterial pathogens including *B. pseudomallei* (22). To do this, they used phagocytosis, antimicrobial peptides, enzymes, reactive oxygen species (ROS) and neutrophil extracellular traps (NETs) containing extracellular fibers with granular proteins and extracellular DNA (eDNA) (23–25). NETs have a key function in capturing and eradicating large pathogens that resist phagocytosis (26, 27). In a previous study, *B. pseudomallei* efficiently induced NETosis, with large amounts of NET-related components to entrap and efficiently kill *B. pseudomallei* but this failed to limit bacterial spreading and inflammation during *B. pseudomallei*-induced sepsis (28). Likewise, impaired NETs in diabetes mellitus patients were possibly correlated with susceptibility to clinical melioidosis and other forms of sepsis (29). In addition, not only host factors influenced NET formation but also bacterial components including *B. pseudomallei* type 3 secretory system (T3SS) and capsular polysaccharide (29). Furthermore, soluble ligands such as lipopolysaccharide (LPS) are well established to stimulate aggregated and cloudy NETs (30). The challenge of eradicating *B. pseudomallei* biofilm has generated considerable interest in NET activation and killing competence but to date has yielded very limited data. Therefore, we aimed to extend knowledge of the association between *B. pseudomallei* biofilm and NET formation. Further understanding of *B. pseudomallei* biofilm and NETs may provide further explanation of the complicated outcome of melioidosis pathogenesis and novel approaches to clinical therapy.

## Materials and methods

### Ethics approval

Blood was collected from healthy donors from the Blood Bank, Srinagarind hospital, Faculty of Medicine, Khon Kaen University, Thailand. This study was approved by Khon Kaen University Ethics Committee for Human Research (Reference No. HE631099).

### Neutrophil isolation

Peripheral blood was obtained from healthy donors using lithium-heparin vacutainers (Greiner Bio-One, Chonburi, Thailand). Neutrophils were isolated as previously described (31). In brief, whole blood was mixed with HetaSep solution (STEMCELL-Technologies, Vancouver, Canada) at a ratio of 5:1 and incubated at 37°C for 30 min. Then, the upper layer containing all nucleated blood cells was taken and gently layered on top of Ficoll-Paque solution (GE Healthcare, Little Chalfont, UK) at a ratio of 1:1, followed by centrifugation at 500 g for 30 min. After peripheral blood mononuclear cells (PBMC), plasma and Ficoll-Paque solution were carefully discarded, the neutrophil pellet was resuspended in 1 mL RPMI 1640 complete medium (RPMI 1640 (Gibco, Life Technologies, Paisley, UK) with 10% fetal bovine serum (FBS) (Gibco, Life Technologies, Paisley, UK)). Hypotonic ammonium chloride lysis buffer was applied to eliminate residual erythrocytes for 4 min, followed by centrifugation at 400 g for 3 min. the neutrophil pellet was then resuspended in RPMI 1640 complete medium and checked for neutrophil purity (> 95%) and viability (> 95% required for further investigations).

### Bacterial strains and growth conditions

Three *B. pseudomallei* strains, H777, a moderate biofilm producer; M10, a biofilm-defective mutant of H777 (inactivated *bpsl0618*) and C17, a biofilm-complemented derivative of M10 (*bpsl0618* restored) (13, 14) were used in this study (**Table 1**). Each *B. pseudomallei* strain from -80°C glycerol stock was streaked on Luria-Bertani (LB) agar plate with appropriate antibiotics and incubated at 37°C for 48 h. A single colony of each strain was sub-cultured into 5 mL of LB broth and incubated at 37°C with 200 rpm for 16-18 h. The bacterial culture was adjusted to an optical density (OD) at 600 nm of 0.1 for 2% inoculums (v/v) in LB broth at 37°C at 200 rpm to obtain the mid-log phase for neutrophil co-cultivation.

**Table 1:** *B. pseudomallei* strains.

*B. pseudomallei*	Characteristics	Source/Description	Antibiotics supplemented	References
H777	Clinical isolate, moderate biofilm producer	Blood from melioidosis patient, Thailand	None	(20)
M10	Biofilm defect mutant of H777	Tn5-0T182 mutagenesis used to inactivate *bpsl0618* (a sugar transferase gene)	Tetracycline 50 µg/mL	(20)
C17	Biofilm complemented of M10	Function of *bpsl0618* restored	Tetracycline 50 µg/mL Chloramphenicol 30 ug/mL	(14)

### Neutrophil mediated-killing

Neutrophils (5 × 10^5^ cells) in 500 µL were added into wells of a 48-well tissue-culture plate and incubated at 37°C for 90 min, 5% CO_2_ and pretreated or not with 10 μg/mL cytochalasin D (Sigma-Aldrich, Saint Louis, USA) for 30 min to prevent phagocytosis as previously described (29). Planktonic *B. pseudomallei* H777, M10 and C17 from mid-log phase cultures were added at a multiplicity of infection (MOI) of 10 and the plates further incubated for 90 min. The percentage of remaining bacteria in each well was then enumerated by serial dilutions and a drop plate technique on LB agar at 37°C for 24 h. The effect of cytochalasin D/dimethyl sulfoxide (DMSO) on neutrophil and bacterial survival was also determined in parallel assays.

### Visualization of NETs

NETs stimulated by *B. pseudomallei* were visualized as previously described (24). Briefly, neutrophils (5 × 10^5^ cells/well) were seeded onto BSA-coated cover slips (1% BSA (Sigma-Aldrich, Saint Louis, USA) in phosphate-buffered saline (PBS), pH 7.4) in 24-well plates at 37°C, 5% CO_2_ for 30 min to allow for cell attachment. Planktonic *B. pseudomallei* H777, M10 and C17 were washed twice with PBS before being incubated with the neutrophils at MOI of 10 in parallel with 50 nM of phorbol 12-myristate 13-acetate (PMA) (Sigma-Aldrich, Saint Louis, USA) (positive control) and untreated controls for 90 min. In addition, neutrophils pretreated with 1 unit/mL DNase I for 15 min prior to adding *B. pseudomallei* H777 were also used. After centrifugation at 1,800 g for 10 min, the cells on the cover slips were fixed with 4% paraformaldehyde for 15 min at room temperature and washed twice with PBS. Cells were permeabilized with 0.5% Triton X-100 for 1 min followed by nonspecific blocking using 1% BSA in PBST (PBS with 0.1% Tween^®^ 20) for 30 min. The samples were stained with the primary rabbit anti-myeloperoxidase antibody (anti-MPO, ab109116, 1:100 dilution, Abcam, Cambridge, UK) for 1 h, washed 3 times with PBST followed by addition of donkey anti-rabbit IgG HL antibody (Alexa Fluor^®^ 647, 1:500 dilution, Abcam, Cambridge, UK). In parallel, mouse anti-neutrophil elastase antibody (anti-NE, ab255935, 1:250 dilution, Abcam, Cambridge, UK) was added for 1 h followed by goat anti-mouse IgG HL antibody (Alexa Fluor^®^ 488, 1:500 dilution, Abcam, Cambridge, UK). DNA scaffolds of NETs were observed after staining with 1 µg/mL of 4′,6-diamidino-2-phenylindole (DAPI) (Thermo scientific, Rockford, USA) for 5 min. Features of the NETs were examined on two different cover slips per treatment at 63× magnification for least 15 fields/cover slip under a confocal laser scanning microscope (LSM-500 and LSM 800; Zeiss, Germany).

To visualize *B. pseudomallei* entrapment, the co-cultured neutrophils and *B. pseudomallei* strains on cover slips were fixed with 4% paraformaldehyde, stained with Giemsa and observed at 100× magnification (Axio, Carl Zeiss, Jena, Germany). Percentage of each NET formation type stimulated by each *B. pseudomallei* strain were quantified (32).

### Quantification of extracellular DNA from *B. pseudomallei* stimulated NETs

Amount of eDNA from NETs was quantified as previously described (33). Briefly, purified neutrophils (5 × 10^5^ cells) in 48-well plates were activated with either *B. pseudomallei* H777, M10 or C17 at MOI 10 for 90 min or with PMA. Subsequently, the mixture was treated with 1 unit/mL micrococcal nuclease (MNase) (Sigma-Aldrich, Saint Louis, USA) for 15 min at room temperature to liberate NET-DNA. After centrifugation at 1800 g for 10 min, the eDNA in the supernatant was quantified with a QuantiFluor^®^ dsDNA system (Promega, Madison, WI, USA) (excitation: 504 nm/emission: 531 nm) using a fluorometer (Varioskan Flash Multimode Reader, Singapore) with SkanIt Software 2.4.3 RE for Varioskan Flash. A standard curve for eDNA was constructed using lambda DNA to quantify eDNA in each sample.

### NET-mediated bacterial killing

NETs bactericidal against *B. pseudomallei* H777, M10 and C17 were examined as previously (29). Neutrophils (5 × 10^5^ cells) in 48-well plates were pretreated with either 10 μg/mL cytochalasin D for 30 min, 1 unit/mL DNase I for 15 min or both agents at 37°C prior to addition of *B. pseudomallei* H777, M10 or C17 at MOI of 10. After incubation for a further 90 min, the mixture in each well was 10-fold serially diluted for bacterial survival enumeration using a drop plate technique on LB agar plates. This experiment was performed in triplicate on three independent occasions.

### Statistical analysis

Statistical analysis was performed using GraphPad Prism version 8.3.4 software (GraphPad, San Diego, CA). Quantities of intracellular bacterial surviving and eDNA were compared among the three experimental groups using two-way ANOVA followed by Tukey’s *post hoc* test for comparisons between pairs. A statistically significant difference was considered as follows: *p < 0.05, **p < 0.01, ***p < 0.001 and ****p < 0.0001.

## Results

### *Burkholderia pseudomallei* H777 and C17 better persist against non-phagocytic killing

**Figure 1:**
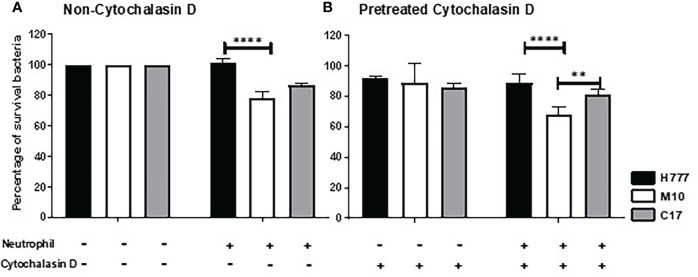
*Burkholderia pseudomallei* H777 significantly better withstood neutrophil-mediated killing with or without the presence of cytochalasin D than did M10, the strain lacking functional *bpsl0618*. The extent of neutrophil-mediated killing was assessed after 90 min co-cultivation of the bacteria with neutrophils untreated **(A)** or pretreated **(B)** with cytochalasin D. Percentage of *B. pseudomallei* cells surviving were determined from 3 independent experiments. Data are represented as mean ± standard deviation. Asterisks denote statistical significance (**p < 0.01, ****p < 0.0001).

To determine whether the *B. pseudomallei* biofilm phenotype could influence bacterial survival from neutrophil mediated killing either all neutrophil eradication strategies or non-phagocytosis, we pre-treated neutrophils with the phagocytosis inhibitor, cytochalasin D before co-cultured with MOI 10 of *B. pseudomallei* H777 or C17 strains containing *bpsl0618* compared to M10 strain with *bpsl0618* defected mutant for 90 min. Significantly more *B. pseudomallei* H777 cells (101.36% of initial number) persisted against untreated neutrophils than was the case for M10 (78.55%). Likewise, the percentage of *B. pseudomallei* H777 cells surviving was significantly higher after encountering neutrophils pretreated with cytochalasin D (88.52%) than that of M10 (68.04%) (*p* < 0.0001) (**Figures 1A, B**). Meanwhile, *B. pseudomallei* C17, the *bpsl0618-*complemented derivative of M10, displayed restored bacterial survival against neutrophil functions. This evidence clearly indicated the better persistence of *B. pseudomallei* H777 and C17 harboring *bpsl0618* against neutrophil bactericidal activities involving a non-phagocytosis mechanism.

### *Burkholderia pseudomallei* H777, M10 and C17 stimulate diverse morphotypes of NETs

**Figure 2:**
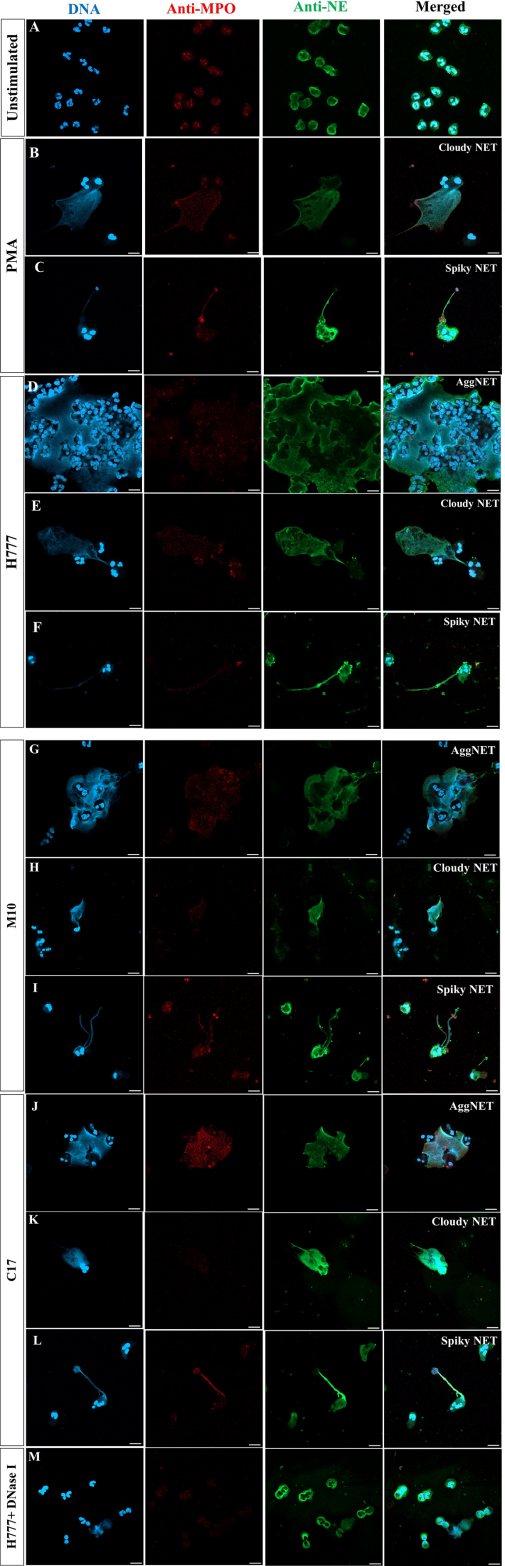
NET formation visualization and effect of DNase I degradation. **(A)** Unstimulated neutrophils; **(B, C)** neutrophils stimulated with PMA; **(D–F)** *B. pseudomallei* H777 (*bpsl0618* wild type); **(G–I)** *B. pseudomallei* M10 (*bpsl0618* mutant); **(J–L)** *B. pseudomallei* C17 (*bpsl0618* complemented); **(M)** *B. pseudomallei* H777 in the presence of 1 unit/mL DNase I. Neutrophils were stained with DAPI (blue), anti-MPO (Alexa Fluor 647 conjugated, red) and anti-NE elastase (Alexa Fluor 488 conjugated, green). Magnification = 63×; scale bars = 10 µm.

To further investigate the ability of *bpsl0618* to facilitate persistence of *B. pseudomallei* against non-phagocytosis mechanisms of human neutrophils, NET formation morphology was examined. Neutrophils were co-cultured with *B. pseudomallei* H777, M10 and C17 in company with PMA as a positive control. NET formation visualization used three NET markers; DAPI to detect released DNA backbone, anti-MPO and anti-NE antibodies. **Figure 2A** shows confocal fluorescence micrographs of unstimulated, intact neutrophils with nuclei, myeloperoxidase and elastase within the cytoplasm (**Figure 2A**). PMA-stimulated neutrophils produced mainly “cloudy” NETs (cloud-like DNA pattern) following the collapse of the nucleus and cell-membrane rupture (**Figure 2B**). Another appearance that NETs could adopt was “spiky” (released DNA traps with fiber-like structure) with colocalized myeloperoxidase (red) and neutrophil elastase (green) with protruding DNA (blue) after activation with PMA (**Figure 2C**). The presence of *B. pseudomallei* H777 produced mainly “aggregated” NETs (high-density aggregation of netting neutrophils) followed by cloudy and spiky NETs (**Figures 2D–F**; **Table 2**). In addition, extrusion of DNA traps from several cells appeared broad and blurry in the extracellular environment. Cells of *B. pseudomallei* M10 mainly led to the production of spiky NETS followed by aggregated and cloudy NETs (**Figures 2G–I**; **Table 2**), whereas *B. pseudomallei* C17 could elicit all types of NETs (**Figures 2J–L**; **Table 2**). Interestingly, C17 led to the appearance of aggregated (34%), cloudy (15%) at spiky NETs (51%) at proportions intermediate between those produced by H777 and M10 (**Table 2**). Thus, the appearance of NETs varies according to the presence or absence of functional *bpsl0618* in *B. pseudomallei* strains stimulating them. The exceedingly high neutrophil accumulation was observed in the aggregated NETs but not in the cloudy or spiky NETs. DNase I treatment of *B. pseudomallei* H777-infected neutrophils resulted in diminished extracellular DNA, MPO and NE suggesting that the released materials from stimulated neutrophils were composed largely of DNA (**Figure 2M**).

**Table 2:** The percentage of NET formation after co-culture with *B. pseudomallei* H777, M10 and Cl7.

Morphotype of NETs formed	Bp H777	Bp M10	Bp C17
**Aggregated**	29/48 (60%)	11/40 (27%)	14/41 (34%)
**Cloudy**	11/48 (23%)	3/40 (8%)	6/41 (15%)
**Spiky**	8/48 (17%)	26/40 (65%)	21/41 (51%)

The data are from 3 independent experiments (n≥40).

**Figure 3:**
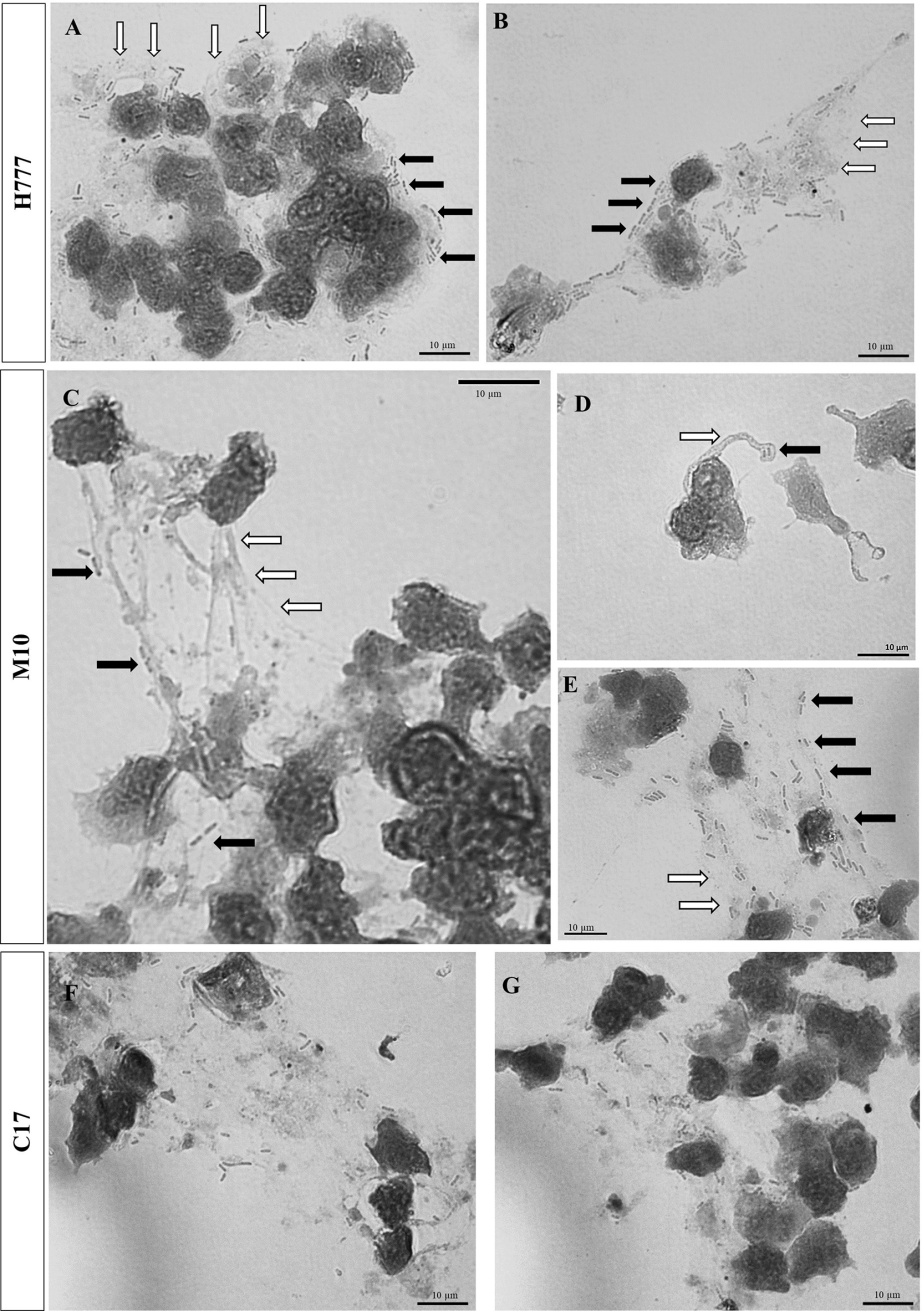
Bright-field images showing that *B. pseudomallei* cells entrapped within NETs after being encountered by neutrophils at MOI of 10 for 90 min and stained with Giemsa dye. **(A, B)** *B. pseudomallei* H777 (rod shape, black arrow) trapped by aggregated NET (gray area, white arrow); **(C)** *B. pseudomallei* M10 entrapped within NETs, **(D)** *B. pseudomallei* M10 entrapped in spiky NETs; **(E)** *B. pseudomallei* M10 trapped within aggregated NETs. **(F, G)** *B. pseudomallei* C17 entrapped within aggregated NETs. Scale bars = 10 µm.

Additionally, bright-field microscopic images during NET formation confirmed that *B. pseudomallei* were entrapped within NETs (**Figure 3**). Cells of *B. pseudomallei* H777 were entrapped within aggregated NETs and liberated NET components or extracellular DNA components (extracellular hazy-gray zone) (**Figures 3A, B**). Moreover, *B. pseudomallei* M10 cells were either confined by the sticky DNA within the boundary of complex spiky NETs (**Figure 3C**), hooked by single extracellular DNA fibers (**Figure 3D**) or entrapped with a DNA cloud of aggregated NETs (**Figure 3E**). Similarly, *B. pseudomallei* C17 were restrained by aggregated NETs (**Figures 3F, G**). These phenomena demonstrated that *B. pseudomallei* could potentially induce NET formation in different manner depending on their possession or not of *bpsl0618*. Extracellular NET components released from stimulated neutrophils were confirmed to capture *B. pseudomallei* cells.

### *Burkholderia pseudomallei* H777 and C17 trigger a higher level of NET DNA release than M10

**Figure 4:**
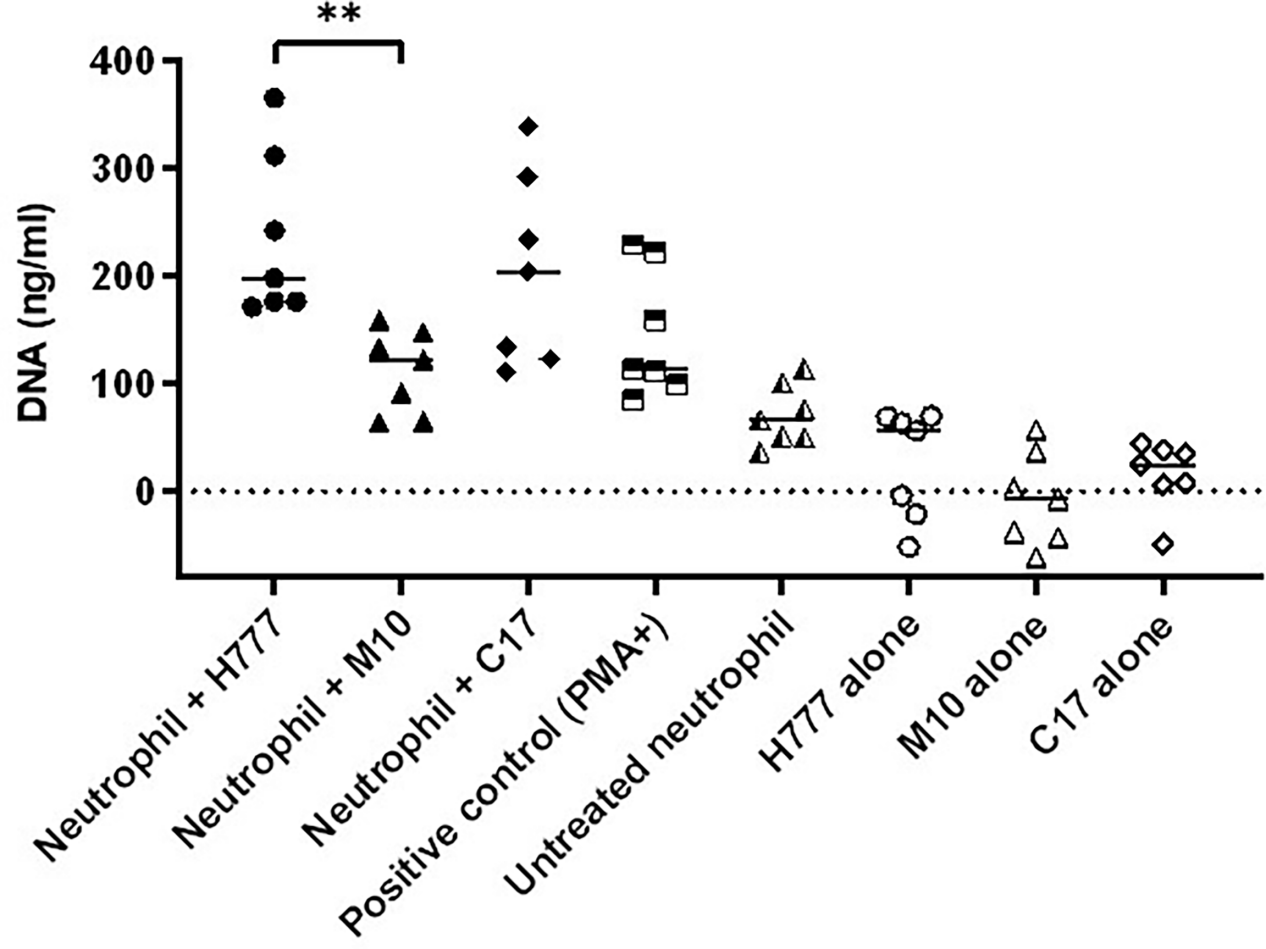
*B. pseudomallei* H777 (*bpsl0618* wild type) and C17 (*bpsl0618* complemented) induced greater release of extracellular DNA from neutrophils than did M10 (*bpsl0618* defect mutant). Neutrophils were stimulated with either *B. pseudomallei* at the MOI of 10 or 50 nM PMA followed by MNase treatment in supernatant. The DNA was quantified in 3 independent experiments (n = 7). Data are represented as mean ± standard deviation. Asterisks denote statistical significance (**p < 0.01).

The quantity of released DNA from NETs activated by *B. pseudomallei* was compared between bacterial strains with or without a functional *bpsl0618* gene. DNA was measured after micrococcal nuclease (MNase) digestion. The results showed that neutrophils activated by *B. pseudomallei* H777 liberated significantly higher quantities of DNA in traps (234.62 ng/mL) than did *B. pseudomallei* M10 (111.87 ng/mL) (*p* ≤ 0.01) (**Figure 4**). While *B. pseudomallei* C17 elicited release of intermediate amounts of NET DNA (205.43 ng/mL). These results suggest that the *B. pseudomallei bpsl0618* gene encoding a sugar transferase is associated with greater production of NETs and NET DNA.

### *Burkholderia pseudomallei* H777 and C17 cells tolerate NET-mediated killing

**Figure 5:**
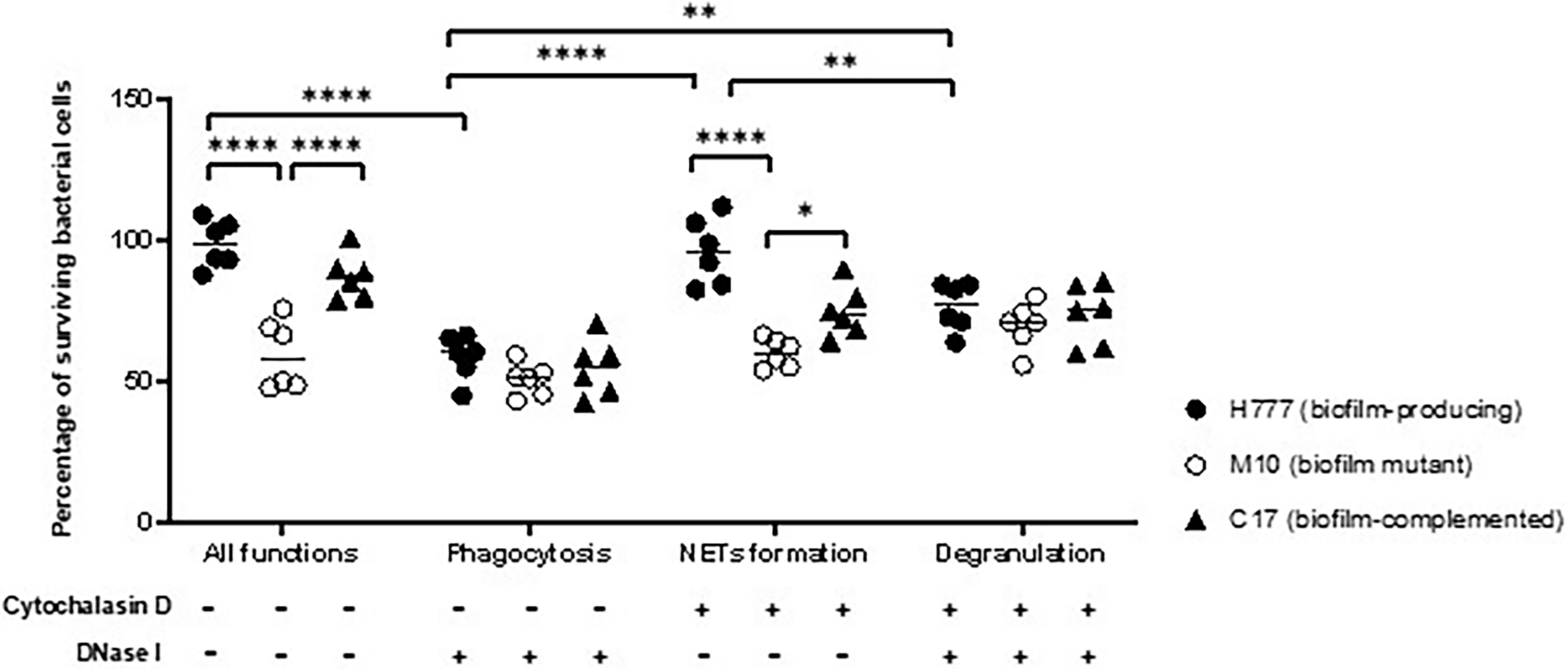
NET formation was insufficient to kill *B. pseudomallei* cells expressing *bpsl0618*. Co-cultivation of purified neutrophils and three strains of *B. pseudomallei* was done in the presence or absence of 10 μg/mL cytochalasin D and/or 1 unit/mL DNase I for 90 min. Percentage of cells surviving in each of the three strains of *B. pseudomallei* is shown in results from 3 independent experiments (n = 6). Data are represented as mean ± standard deviation. Asterisks denote statistical significance (*p < 0.05, **p < 0.01, ****p < 0.0001).

Following NET stimulation, the fate of each *B. pseudomallei* strain was monitored against neutrophil-mediated killing mechanisms: phagocytosis, NET-mediated killing and degranulation. Neutrophils were treated or not with either or both of DNase I and cytochalasin D. The results revealed that cells of *B. pseudomallei* H777 and C17 could persist against neutrophil-mediated killing (untreated neutrophils) (98.77 and 87.17%) but that survival was much lower in the *bpsl0618*-inactivated mutant, M10 (59.57%). Similar percentages of *B. pseudomallei* H777 and C17 could survive NETosis (pretreatment of neutrophils with cytochalasin D) (96.24 and 74.76%, respectively) while survival of M10 cells was significantly lower (59.95%) (**Figure 5**). However, all three strains were almost equally susceptible to phagocytic killing by neutrophils pretreated with DNase I: survival rates were 58.59% for H777, 50.58% for M10 and 54.65% for C17 cells. These data highlighted the crucial role of the *bps0618* gene on *B. pseudomallei* biofilm formation ability that endurance against NET-mediated killing but not of phagocytosis. However, in the presence of both cytochalasin D and DNase I, leaving neutrophils only the option of degranulation-mediated killing, all three bacterial strains were equally susceptible. These results emphasized that NET stimulation by *B. pseudomallei* is insufficient to eradicate *B. pseudomallei* strains with the ability to form biofilm and that the presence of the *bpsl0618* gene assists survival against NET-mediated bacterial killing.

## Discussion

Biofilm-associated *Burkholderia pseudomallei* infections are often associated with high incidence of relapse of melioidosis (10), resulting in poor clinical outcomes (9). The possibility of bacterial biofilms skewing host immune balance remains to be elucidated (19). Production of NETs is well recognized as one of the neutrophil-mediated killing mechanisms to capture and kill pathogens capable of biofilm formation and resistant to phagocytosis. To date, a number of studies have focused on bacterial strategies to escape from NETs and influence the outcome of bacterial infections (34). Furthermore, evasion of NETs has been seen in respiratory pathogens, thus facilitating pathogen proliferation and dissemination (25). *B. pseudomallei* strains with the ability to produceT3SS and capsular polysaccharide were better able to evade NETs (29). The formation of NETs stimulated by presence of *B. pseudomallei* does not prevent bacterial dissemination and inflammation (28). To the best of our knowledge, limited information is available on the association between *B. pseudomallei* biofilm and NET formation and the consequences for bacterial survival. Our study offers further evidence of the effects of *B. pseudomallei* with the biofilm formation capability on neutrophil- and NET-mediated killing using planktonic *B. pseudomallei* H777, a biofilm wild type compared to that of the M10 strain, a biofilm-defective mutant of H777 (inactivated *bpsl0618*, a sugar transferase gene) and C17, a biofilm-complemented derivative of M10. The biofilm formation ability of these three strains was previously determined in our laboratory (13, 14). Remarkably, we have shown that *B. pseudomallei* biofilm phenotypes can withstand non-phagocytotic activities of neutrophils. The lowest survival rate in our experiments was seen in the *B. pseudomallei* biofilm-defective strain, emphasizing the susceptibility of the mutant to NETs and phagocytosis. Additionally, the distinct morphological types of NET (aggregated, cloudy and spiky) were first visualized based on presence or absence of a *bpsl0618* gene in *B. pseudomallei*. The presence of this gene, encoding sugar transferase, was correlated with higher levels of extracellular DNA extruded from stimulated neutrophils. Notably, the twice higher level of DNA from NET stimulated by *B. pseudomallei* H777 and C17 than that of M10 indicated a better potential biofilm formation capability of this pathogen to stimulate NET formation. Nevertheless, NET formation failed to eradicate *B. pseudomallei* H777 and C17 but had a considerable impact on M10, the biofilm-defective mutant. However, all three strains were equally susceptible to phagocytotic killing by neutrophils. This study highlighted the possession of *bpsl0618* that was previously demonstrated to be associated with the ability of *B. pseudomallei* to form biofilm (13, 14) and may allow persistence of these bacteria despite the fact that their presence stimulates production of NETs, one of the key innate eradication mechanisms employed by the body.

Our findings are consistent with previous results in which *B. pseudomallei* showed genuine resistance to neutrophil killing (22). Furthermore, the persistence of *B. pseudomallei* H777 and C17 against neutrophil- and NET-mediated killing are consistent with the findings of de Jong and colleagues (28), who demonstrated that *B. pseudomallei* potentially induced NET formation in humans based on the finding of NET-related components in melioidosis patients. Even so, presence of NETs hardly protects against bacterial dissemination.

Pathogenic bacteria have several approaches to protect themselves from NET capture and killing. These include using polysaccharide capsules, biofilm formation, altering electric charge of cell surface, inhibition of NETs by peptidase and DNase secretion and inhibiting ROS production (34). Both the biofilm-competent *B. pseudomallei* H777 harboring *bpsl0618* and C17, the *bpsl0618-*complemented strain, efficiently induced NETs but could survive despite this. This are in line with previous findings (29), which demonstrated the crucial roles of T3SS and capsular polysaccharide produced by *B. pseudomallei* in evasion of NET killing mechanisms. The impact of these mutants that interrupt the bacterial cellular process represented known virulence factors of *B. pseudomallei* during NET formation. Similar findings have been noted for non-typeable *Haemophilus influenza* biofilms: despite being entrapped within NETs, these cells could survive both phagocytic and extracellular neutrophil killing resulting in increased numbers of bacterial cells. Consequently, the persistence of *H. influenza* correlated with chronic and recurrent otitis media infections (35). Based on our results, it is at least hypothetically possible that *B. pseudomallei* biofilm may stimulate NET production but escape from the bactericidal activities of NETs leading to bacterial persistence and chronic infection or relapsing melioidosis.

NET formation is an alternative defense mechanism assisting neutrophils to kill particularly large pathogens (26). NETs principally immobilize pathogens to prevent microbial spreading. The antimicrobial activity of NETs is exerted through direct contact of histones, antimicrobial peptides, neutrophil elastase, myeloperoxidase (MPO) and cathepsin G4 with the entrapped pathogens. The negatively charged DNA that traps and immobilizes pathogens manifest as sticky fibers (30). Additionally, the molecular mechanism of NET formation upon *B. pseudomallei* infection was indicated that NADPH oxidase was crucial but not phosphatidylinositol-3 kinase, mitogen-activated protein kinases, or Src family kinase signaling pathways (29).

In this study, we have illustrated the appearances of different types of NETs induced *in vitro* by several *B. pseudomallei* strains: biofilm wild type (H777), biofilm-defective mutant (M10) and biofilm-complemented (C17). Aggregated NETs were mainly found after exposure of neutrophils to *B. pseudomallei* biofilm wild type, less commonly for the biofilm-defective mutant but this type of NET was restored when neutrophils were exposed to the complemented strain. This is in complete agreement with the immunofluorescence images of LPS-stimulated NETs resulting in clumps of multiple neutrophils, both alive and dead, and bacteria (30). Large NETs of this type contain excess enzymes, including nuclease, which is vital for bacterial clearance. Additionally, aggregated NETs also contain both inflammatory mediators and pro-inflammatory cytokines and chemokines which support resolution of neutrophil-driven inflammation (30). Furthermore, the aggregated NETs are responsible to limit neutrophil inflammation by degrading cytokines and chemokines and disrupting neutrophil recruitment and activation in gout was demonstrated (36). Therefore, the higher proportion of aggregated NETs induced by *B. pseudomallei* biofilm phenotypes compared to that of the biofilm mutant, might be associated with a pathogen-survival stragegy. The bright-field images in our study have shown that *B. pseudomallei* H777 cells were entrapped within the boundaries of aggregated NETs in which were present myeloperoxidase and neutrophil elastase. Despite this, the NETs were unable to kill the entrapped cells. In a similar way, non-typable *H. influenzae* cells found within biofilm-associated chronic otitis media were not killed by NETs (37, 38).

Single “cloudy” NETs were commonly induced by PMA and LPS (30, 39). The neutrophils exhibited round nuclei and ruptured membranes with chromosomal DNA extruded giving a cloudy appearance. Moreover, NETosis-driven cloudy NETs may result in extracellular release of danger-associated molecular patterns (DAMPs) such as the necrotic cell-death pathway, leading to overwhelming inflammation and pathological immune responses (39). Cloudy NETs were most strongly induced by biofilm-producing H777, with the *bpsl0618*-complemented C17 strain inducing levels intermediate between H777 and the biofilm-defective strain, M10. This may imply a correlation of the biofilm phenotype with pathological immune response.

The fiber-like structure of “spiky” NETs exocytosed from intact neutrophils implied that the neutrophils remain viable (30). Spiky NETs were most frequently triggered by the M10 strain, which lacks *bpsl0618*. Survival of neutrophils was noted in the severe Gram-positive infection reported by Yipp and colleagues (40). They showed that the NETing neutrophils turned to anuclear cytoplasts and continued to phagocytose and prevent bacterial dissemination.

Our study has some limitations. We conducted experiments only *in vitro* and using only neutrophils. However, it may provide information concerning the direct consequence of *B. pseudomallei* biofilm on NET formation. The study of cytokines during NET formation upon encountered with *B. pseudomallei* would strengthen our study to disclose the impact of the bacterial biofilm on NET formation and melioidosis pathogenesis. In addition, planktonic cells of *B. pseudomallei* biofilm phenotype, mutant and complemented strains were used in this study to ensure comparable bacterial numbers. The role of biofilm-associated phenotypes were verified for their characters and correlated in cellular pathogenesis (14). The similar bactericidal efficacy of NETs on *Streptococcus suis* biofilms and planktonic cells were illustrated (27). Furthermore, the introduction of the empty vector introduced into the *B. pseudomallei* M10 may discard the unknown effect of the vector on the NET morphotypes.

Taken together, we have first illustrated various morphotypes of NETs (aggregated, cloudy and spiky) triggered by exposure to *B. pseudomallei* strains with or without the *bpsl0618* gene, a sugar-transferase gene that is associated with the ability to form biofilm. The presence of this gene may transform planktonic *B. pseudomallei* cell surfaces, thus boosting production of NETs as double-edged swords that can shield *B. pseudomallei* cells rather than eradicate them. This would facilitate pathogen persistence leading to chronic infection or relapse of melioidosis. Our findings provide novel information on the encounters between biofilm-associated *B. pseudomallei* and NETs that extend knowledge and may inform management of chronic or relapsing melioidosis.

## Data availability statement

The original contributions presented in the study are included in the article/Supplementary Material. Further inquiries can be directed to the corresponding author.

## Ethics statement

This study was approved by Khon Kaen University Ethics Committee for Human Research (Reference No. HE631099). Written informed consent for participation was not required for this study in accordance with the national legislation and the institutional requirements.

## Author contributions

MK, SP, KS, and SC contributed to conception and design of the study. MK performed the experiments, the statistical analysis and wrote the first draft of the manuscript. All authors contributed to manuscript revision, read, and approved the submitted version.

## Funding

This work was financially supported by Faculty of Medicine, Khon Kaen University research grant (IN64129).

## Conflict of interest

The authors declare that the research was conducted in the absence of any commercial or financial relationships that could be construed as a potential conflict of interest.

## Publisher’s note

All claims expressed in this article are solely those of the authors and do not necessarily represent those of their affiliated organizations, or those of the publisher, the editors and the reviewers. Any product that may be evaluated in this article, or claim that may be made by its manufacturer, is not guaranteed or endorsed by the publisher.
